# Benign cervical multi-nodular goiter presenting with acute airway obstruction: a case report

**DOI:** 10.1186/1752-1947-4-258

**Published:** 2010-08-10

**Authors:** Anu Sharma, Vijay Naraynsingh, Surujpaul Teelucksingh

**Affiliations:** 1 Faculty of the Medical Sciences, University of the West Indies, St. Augustine, Trinidad & Tobago

## Abstract

**Introduction:**

Benign cervical goiters rarely cause acute airway obstruction.

**Case presentation:**

We report the case of a 64-year-old woman of African descent who presented with acute shortness of breath. She required immediate intubation and later a total thyroidectomy for a benign cervical multi-nodular goiter with no retrosternal tracheal compression.

**Conclusion:**

Benign multi-nodular goiters are commonly left untreated once euthyroid. Peak inspiratory flow rates should be measured via spirometry in all goiters to assess the degree of tracheal compression. Once tracheal compression is identified, an elective total thyroidectomy should be performed to prevent morbidity and mortality from acute airway obstruction.

## Introduction

Benign multi-nodular goiter is a common problem affecting 5% of the general population in non-endemic and 15% [1] in endemic areas. However, the incidence of benign goiter causing acute airway obstruction is as low as 0.6% [2]. Retrosternal goiters account for most of these cases, as growth of the thyroid into the bony rigid thoracic inlet can cause tracheal compression. When a goiter is purely cervical, however, it rarely compresses the trachea to cause obstruction [3]. On review of the literature, only eight reports of cervical goiters causing airway obstruction were found [3-6]. Here, we present the case of a patient with recurrent benign cervical multi-nodular goiter presenting with acute airway obstruction.

## Case presentation

A 64-year-old hypertensive woman of African descent presented to our emergency room with a two-day history of worsening shortness of breath and stridor. She had been aware of a recurrent goiter for over 15 years, having had a partial thyroidectomy 35 years ago for benign multi-nodular disease. Over the past year, she had been experiencing shortness of breath on exertion, generally relieved by rest. However, the period of rest needed to relieve her dyspnea had been increasing in duration. She did not have any hyperthyroid or hypothyroid symptoms and there was no history of fever, dysphagia, pain or hoarseness.

**Figure 1:**
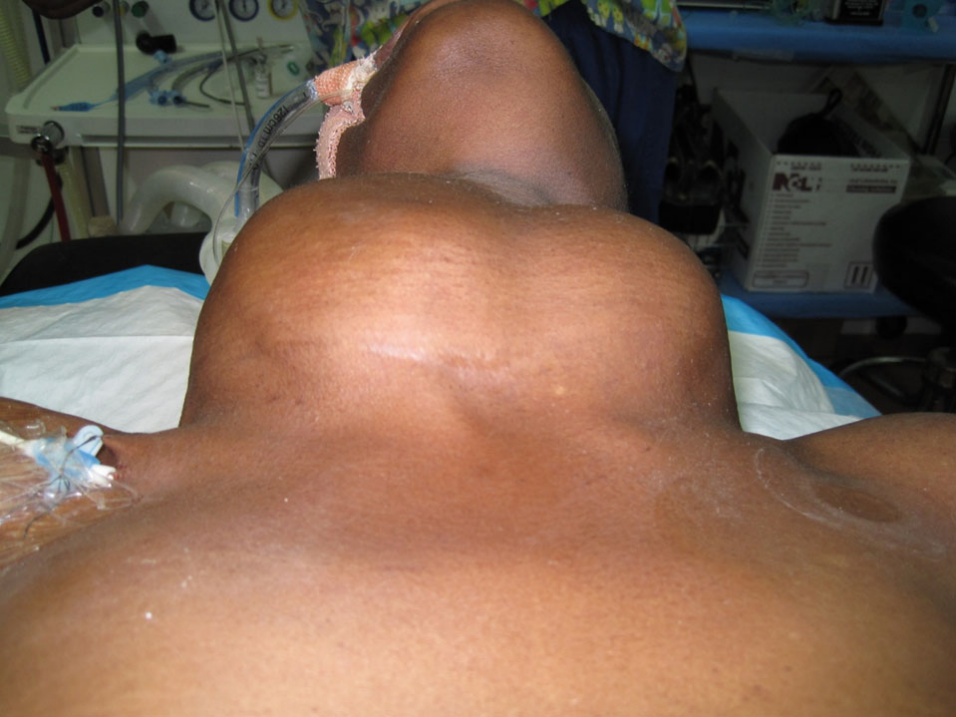
**Large benign multi-nodular goiter**. The figure illustrates the size of the large multi-nodular goiter that our patient presented with. This goiter measured 14×11 cm (right lobe) and 11×8 cm (left lobe). No retrosternal goiter was found on examination. Our patient was intubated and on the ventilator with a central line in place on the right.

On presentation to our emergency department she had marked stridor, tachypnea (32 breaths/minute), tachycardia (120 beats/minute) and blood pressure of 160/95 mmHg. Her pulse oximeter oxygen saturation (spO_2_) was 78% on room air. A large multi-nodular goiter was obvious: right lobe 14×11 cm, left lobe 11×8 cm (Figure 1). All other examinations were normal. She was rushed to the operating theatre for intubation under general anesthesia. A central line was also placed via the right subclavian vein. On intubation, the larynx appeared normal and a 7.5Fr endotracheal tube (ET) was passed easily.

**Figure 2:**
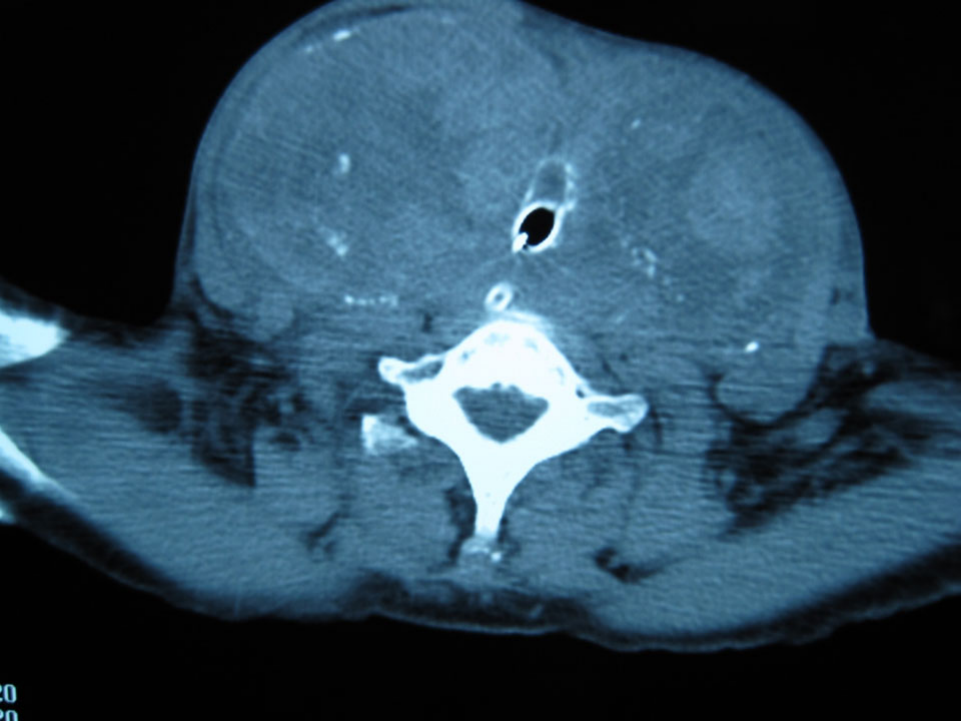
**A computed tomography (CT) scan at the level of C7 showing the endotracheal tube flush with the wall of the trachea**. The diameter of the tracheal lumen measured 7.5 mm with the endotracheal tube *in situ *maintaining its patency. Compare the tracheal diameter in this image with Figure 3.

**Figure 3:**
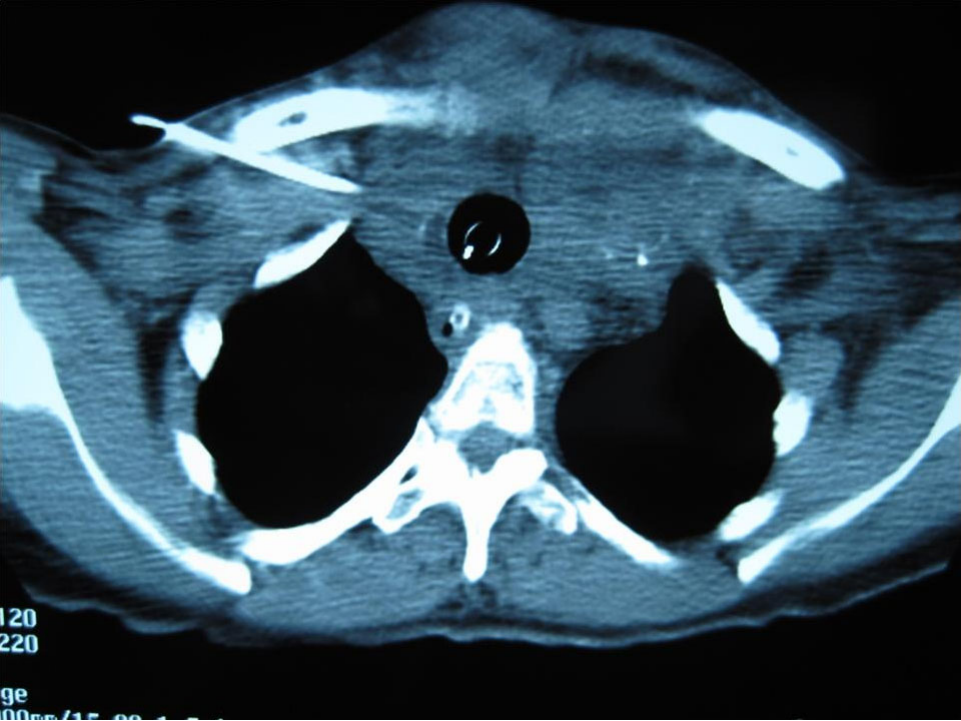
**A computed tomography (CT) scan at the level of T2 showing the endotracheal tube situated within the tracheal lumen**. The tracheal diameter was 2 cm at this level. No retrosternal tracheal compression was evident as compared to Figure 2. There was mild retrosternal extension on the left down to the level of the great vessels. A central venous line was noted on the right.

After intubation, she stabilized and was able to breathe comfortably with the ET *in situ*. She was admitted to the intensive care unit and given propanolol 20 mg orally, three times daily. Her laboratory test results were within normal ranges, with a thyroid-stimulating hormone (TSH) level of 1.4 mIU/L and free T4 level of 1.5 μg/dL. A computed tomography (CT) scan of the neck and thorax showed gross enlargement of both lobes of the thyroid with multiple nodules of varying sizes. There was marked narrowing of the cervical trachea with only the ET maintaining the patency of the airway (Figure 2). There was mild retrosternal extension on the left side down to the level of the origin of the great vessels but the retrosternal trachea was not compressed (Figure 3). The results of an electrocardiogram (ECG) were normal, while the results of an echocardiogram were consistent with hypertensive heart disease with an ejection fraction of 65%.

A total thyroidectomy was performed on the fourth day after admission. The gland was dissected easily with preservation of the recurrent laryngeal nerves and parathyroids. A tracheostomy was placed prophylactically. The trachea was normal with no features of tracheomalacia. She returned to our intensive care unit and recovered with no complications. Her calcium levels did not decline post-operatively. The tracheostomy was removed on day 10 post-operatively. Histology tests confirmed a benign multi-nodular goiter.

## Discussion

Acute airway obstruction has been described extensively for retrosternal benign goiters and thyroid malignancies. Benign cervical goiters causing acute airway obstruction are rare. Reports have been published suggesting acute obstruction to be due to sudden hemorrhage into a cyst, an upper respiratory tract infection causing edema, tracheal collapse or worsening of a medical illness [3,6-9]. In our case, all the above causes had been ruled out.

Jauregui *et al*. [7] suggested upper airway obstruction due to goiter is frequently under-diagnosed. The progressive, insidious growth experienced allows the patient time to compensate for up to 70% of tracheal compression [3]. If specific questions were asked, 45% of patients said they had shortness of breath on either exertion or when in a supine posture [7]. Compromised airflow in patients who are asymptomatic has been proven by spirometry [6-8]. Peak inspiratory flow rates have been shown to be a good indicator for urgent thyroidectomy [9]. Using spirometry as a screening tool, the incidence of upper airway obstruction ranged from 10% to 31% [8]. In all cases, partial or total thyroidectomies were definitive cures to relieve obstruction.

In our patient, spirometry could have identified our patient's compromised respiration but life-saving, urgent intubation was essential. She developed stridor and worsening dyspnea over a two-day period suggesting progressive compromise of the tracheal lumen. In spite of the goiter's large size, no structural tracheal defect was evident at intubation or surgery. This suggests purely mechanical compression of the trachea by the huge goiter within the firm, unyielding cervical fascia, causing her upper airway obstruction.

Her symptoms had been worsening over a year-long period. The slow growth rate of the thyroid gland allows adaptation to extrinsic hypoventilation without acute symptoms [9]. Therefore, in keeping with previous reports, a patient who is asymptomatic with a large multi-nodular goiter should not be taken lightly. The possibility of acute airway obstruction should be discussed, spirometry performed and an elective thyroidectomy offered to patients with large goiters even with lesser grades of compressive symptomatology.

## Conclusion

Benign euthyroid multi-nodular goiters are common. The incidence of acute airway obstruction due to a benign goiter, however, is quite low, with cases due to purely cervical goiters being rare. This has allowed physicians a conservative approach to management. On review of the literature, however, tracheal compression with decreased inspiratory flow rates are found in one-third of cases. The management of benign cervical multi-nodular goiters should include inspiratory spirometry. Once compromised airflow is identified, prophylactic total thyroidectomy should be performed to avoid the dangers of complete airway obstruction.

## Consent

Written informed consent was obtained from the patient for publication of this case report and any accompanying images. A copy of the written consent is available for review by the Editor-in-Chief of this journal.

## Competing interests

The authors declare that they have no competing interests.

## Authors' contributions

ST and AS provided medical assistance to the case and VN provided surgical findings. AS and VN performed the literature search and major contributors to writing the manuscript. ST and VN edited the manuscript. All authors have read and approved the final manuscript.

## References

[B1] Abraham D Singh N Lang B Chan WF Lo CY Benign nodular goiter presenting as acute airway obstruction ANZ J Surg 2007 77 364 367 10.1111/j.1445-2197.2007.04061.x 17497977

[B2] Ríos A RodrÍguez JM Canteras M Galindo PJ Tebar FJ Parrilla P Surgical management of multinodular goiter with compressive symptoms Arch Surg 2005 140 49 53 10.1001/archsurg.140.1.49 15655205

[B3] Sajja LR Mannam GC Sompalli S Simhadri CSR Hasan A Multinodular goiter compressing the trachea following open heart surgery Asian Cardiovasc Thorac Ann 2006 14 416 417 17005891 10.1177/021849230601400514

[B4] Tseng KH Felicetta JV Rydstedt LL Bouwman DG Sowers JR Acute airway obstruction due to a benign cervical goiter Otolaryngol Head Neck Surg 1987 97 72 75 3112689 10.1177/019459988709700114

[B5] Shaha AR Surgery for benign thyroid disease causing trachea-oesophageal compression Otolaryngol Clin North Am 1990 23 391 401 2114603

[B6] Melliere D Saada F Etienne G Becquemin JP Bonnet F Goitre with severe respiratory compromise: evaluation and treatment Surgery 1988 103 367 373 3344488

[B7] Ríos A Rodríguez JM Galindo PJ Cascales PA Blasalobre M Parilla P Spirometric evaluation of respiratory involvement in asymptomatic multinodular goiter with an intrathoracic component Arch Bronchoneumol 2008 44 504 506 10.1016/S0300-2896(08)72122-7 19000515

[B8] Karbowitz SR Edelman LB Nath S Dwek JH Rammohan G Spectrum of advanced upper airway obstruction due to goitres Chest 1985 87 18 21 10.1378/chest.87.1.18 3965261

[B9] Miller MR Pincock AC Oates GD Wilkinson R Skene-Smith H Upper airway obstruction due to goiter: detection, prevalence and results of surgical management QJ Med 1990 74 177 188 2345786

